# Troubleshooting of a left common carotid artery pseudoaneurysm as complication of central venous catheter placement

**DOI:** 10.1007/s00392-021-01871-6

**Published:** 2021-05-18

**Authors:** Juliane Dederer, Peter Fries, Iman Madarati, Michael Böhm, Felix Mahfoud

**Affiliations:** 1 Klinik für Kardiologie, Angiologie und Internistische Intensivmedizin, Universitätsklinikum des Saarlandes, Homburg, Germany; 2 Klinik für Diagnostische und Interventionelle Radiologie, Universitätsklinikum des Saarlandes, Homburg, Germany

Sirs,

An 88-year-old female patient (height: 156 cm, weight: 50 kg) presented in the emergency department with chest pain, dyspnea, and edema of the lower extremities since 2 days. Troponin T, creatinine kinase, and creatinine kinase muscle-brain type were within normal ranges. In the electrocardiogram, there was no evidence of coronary ischemia. Clinical examination and laboratory testing indicated cardiac decompensation with an elevated NT-pro-BNP of 3371 pg/ml (normal range up to the age of 75 years < 623 pg/ml). Mitral valve repair was performed in 2006 because of acute, non-ischemic chordal rupture with a history of paroxysmal atrial fibrillation.

**Fig. 1:**
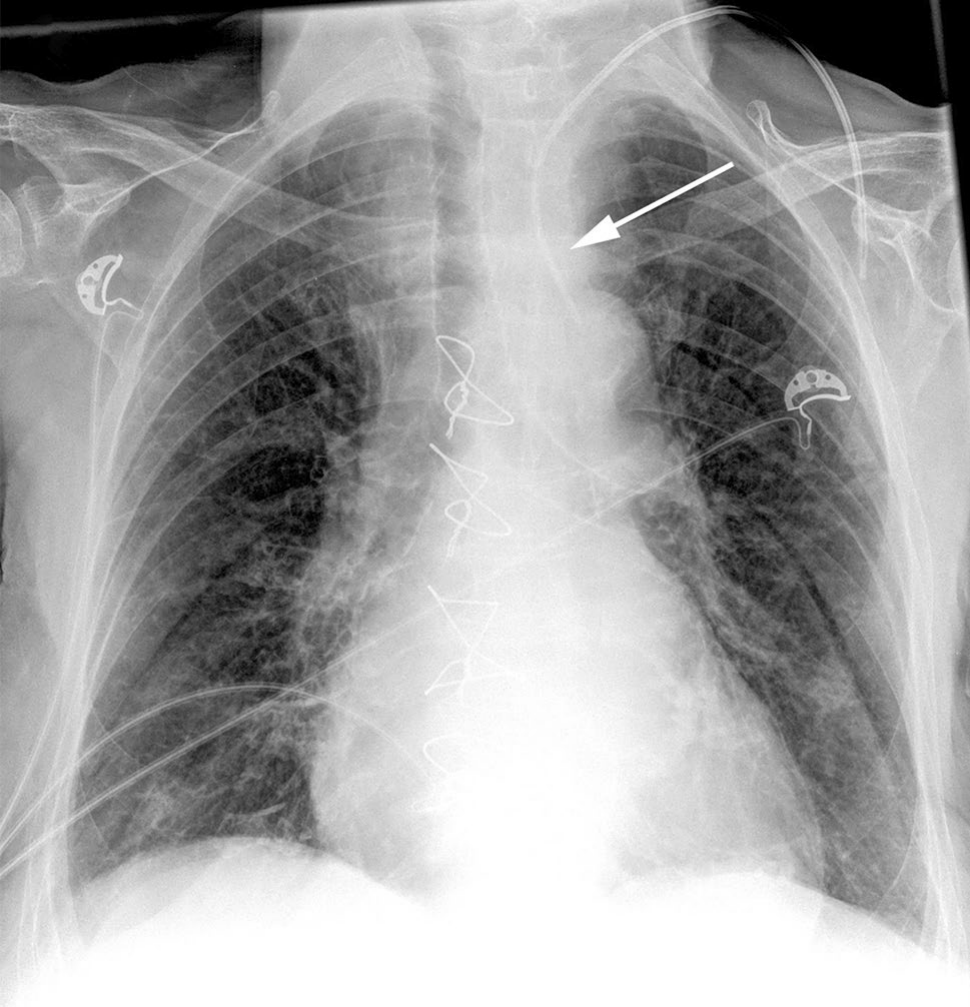
Anterior–posterior chest X-ray demonstrates an atypical course of the inserted central venous catheter (arrow) with projection on the left common carotid artery and its tip on the aortic arch

Oral treatment consisted of torasemide, metoprolol, apixaban, simvastatin, sitagliptin, and pantoprazole. The initial laboratory testing revealed severe hypokalemia (potassium 2.4 mmol/l, normal range 3.5–5.1 mmol/l). For high-dose intravenous potassium substitution, it was planned to insert a central venous catheter (CVC) into the left internal jugular vein. The post-procedural chest X-ray showed an atypical projection of the CVC on the aortic arch and the left carotid artery indicating arterial malpositioning of the CVC (Fig. 1). The CVC was removed immediately, followed by manual compression of the puncture site for 15 min and compression with a sandbag over 1 h. An alternative peripheral access in the right median cubital vein was established and the potassium level was subsequently normalized.

**Fig. 2:**
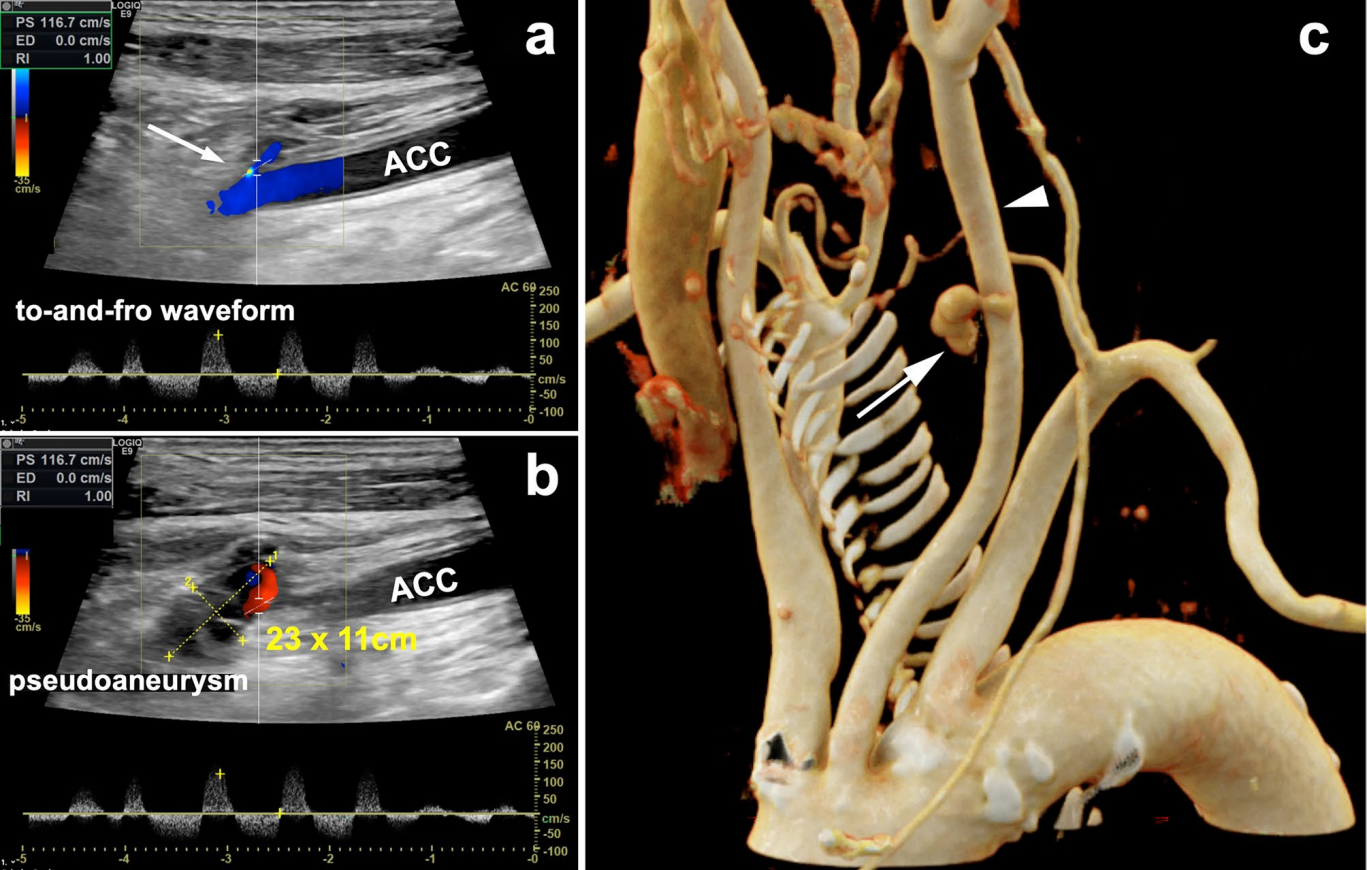
**a** Depiction of the pseudoaneurysm–neck (arrow) outgoing of the common carotid artery (ACC) with to-and-fro waveform by pulse Doppler. **b** Color Doppler showing the pseudoaneurysm sac of 23 × 11 cm with marginal thrombosis. **c** Cinematic rendering reconstruction of the CT angiography in LAO projection shows the pseudoaneurysm (arrow) anterior to the left common carotid artery (arrowhead) originating on the lateral aspect of the vessel wall

Duplex sonography the next day documented a pseudoaneurysm with an extension of 23 × 11 mm in the proximal part of the common carotid artery with the characteristic to-and-fro sign by pulsed Doppler (Fig. 2a, b). The internal and external carotid arteries on both sides were free of stenosis or plaques. A contrast-enhanced computed tomography angiography excluded occult active bleeding (Fig. 2c).

**Fig. 3:**
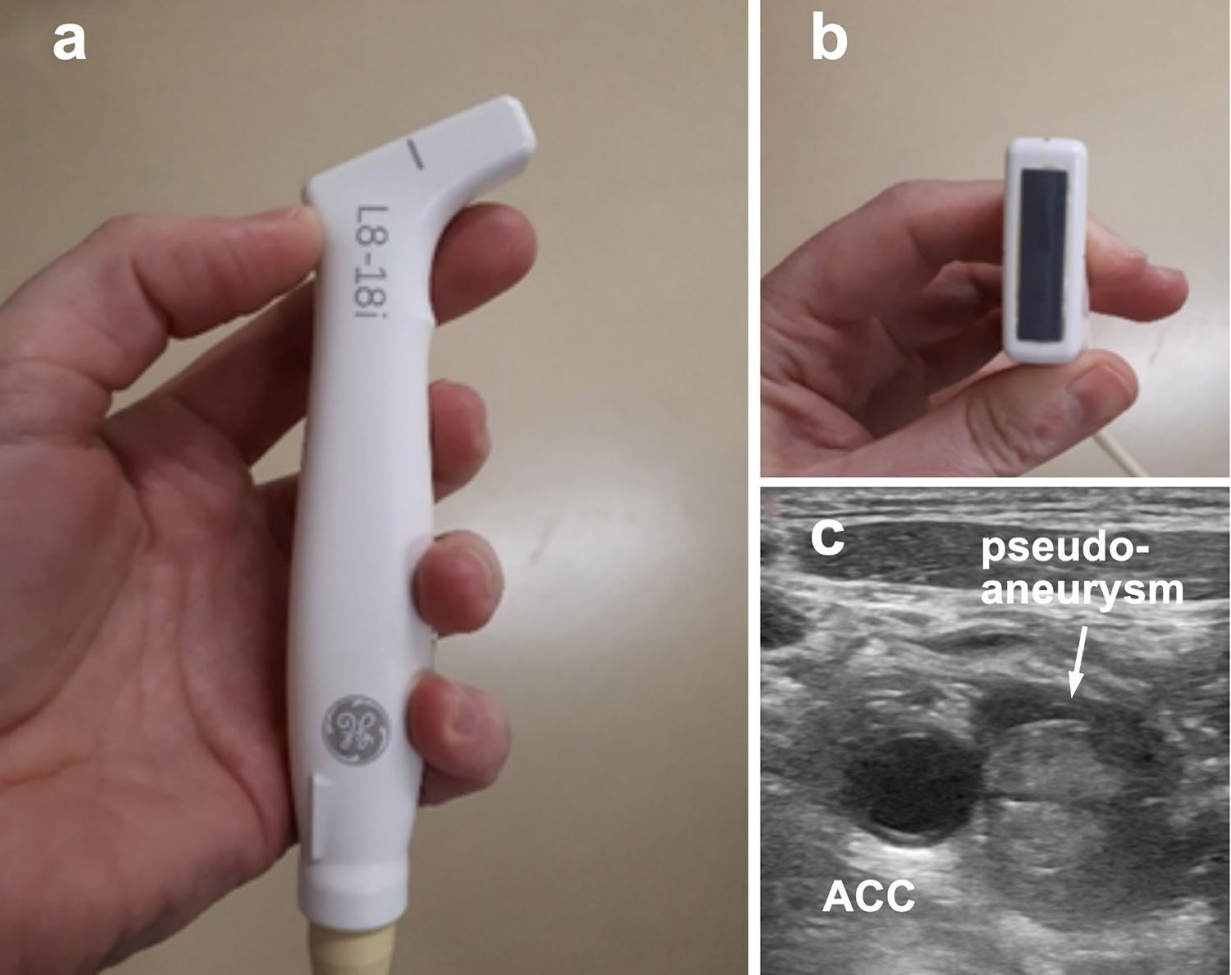
**a**, **b** Shape of the LOGIQ L8-18i finger probe. **c** Complete thrombosis of the pseudoaneurysm (arrow)

Manual compression controlled by duplex sonography was initiated and treatment with apixaban was paused. Due to the higher temporal and spatial resolution, the linear LOGIQ L8-18i finger probe with a high transmission frequency of 15 MHz was used, which also allowed targeted compression of the aneurysm neck due to the small probe size (Fig. 3a, b). The compression lasted for 20 min, during which the common carotid artery was only slightly narrowed without alteration of the intraluminal blood flow. Afterwards, the pseudoaneurysm was completely thrombosed (Fig. 3c). The patient was continuously responsive and adequate, and no pathological neurological findings were present. Duplex sonography the following day, on day 2, and on day 9 showed complete thrombosis of the pseudoaneurysm.

The prevalence of arterial malpositioning associated with central venous access of the internal jugular vein ranges between 0.8 and 19% [1, 2]. The most common vascular complications associated with jugular vein punctures are injuries of the common carotid artery, but also accidental punctures of the brachiocephalic trunk, vertebral artery, thyrocervical trunk, and subclavian artery have been described [3, 4]. In our patient, the so-called ‘overlap phenomenon’ existed, describing the anatomical situation of the internal jugular vein lying in front of the carotid artery [2]. In this situation, accidental arterial puncture is more likely, and may cause thromboembolic stroke, hemorrhage, arteriovenous fistula, dissection, and pseudoaneurysm. The latter occurs more frequently in patients with coagulation disorders and patients requiring oral anticoagulation therapy, respectively [3]. Pseudoaneurysms can quickly increase in volume and size and may compress adjacent structures such as veins and nerves with consecutive neurolysis and venous thrombosis, and can also rupture. Treatment of pseudoaneurysms include surgical and interventional therapies, such as resection, implantation of covered or flow diverting stents, or coiling as well as percutaneous thrombin injection and manual compression [5]. Both interventional and surgical repair may cause severe complications such as major and minor stroke, stent stenosis, and cardio-pulmonary complications and should, therefore, be considered as reserve options [6, 7]. Manual compression of the neck, however, may be challenging, because there is no hard abutment for firm compression of the aneurysm neck and sac. Furthermore, compression of the carotid arteries can be threatening, particularly in the presence of stenosis or plaques as it may cause cerebral hypoperfusion. In the present case, due to the proximal location of the pseudoaneurysm, a compression attempt with the LOGIQ L8-18i finger probe appeared promising and was finally successful, even though the patient was on effective oral anticoagulation (with an anti-Xa level in therapeutic range: 89.2 ng/ml, normal range 34–162 ng/ml).

Importantly, to avoid arterial malpositioning of a CVC, one should get an idea of the location of the internal jugular vein and the carotid arteries and ideally perform an ultrasound-guided puncture. The latter has shown to decrease vascular complications significantly [8]. If the vein is properly filled and not collapsing during inspiration, a controlled puncture of the anterior wall with subsequent catheter placement can be done safely. Especially if the vein lies in front of the artery in close proximity of each other, due to the thin venous wall and its high elasticity, pressure caused by the needle can lead to venous compression and an unintended arterial puncture may follow.
